# Marine Polysaccharides in Pharmaceutical Applications: An Overview

**DOI:** 10.3390/md8092435

**Published:** 2010-09-02

**Authors:** Paola Laurienzo

**Affiliations:** Institute of Polymers Chemistry and Technology, C.N.R.-Via Campi Flegrei, 34-80078 Pozzuoli (Naples), Italy; E-Mail: paola.laurienzo@ictp.cnr.it

**Keywords:** chitosan, alginate, agar, carrageenans, exopolysaccharides, chemical modification, drug delivery, gene delivery

## Abstract

The enormous variety of polysaccharides that can be extracted from marine plants and animal organisms or produced by marine bacteria means that the field of marine polysaccharides is constantly evolving. Recent advances in biological techniques allow high levels of polysaccharides of interest to be produced *in vitro*. Biotechnology is a powerful tool to obtain polysaccharides from a variety of micro-organisms, by controlling the growth conditions in a bioreactor while tailoring the production of biologically active compounds. Following an overview of the current knowledge on marine polysaccharides, with special attention to potential pharmaceutical applications and to more recent progress on the discovering of new polysaccharides with biological appealing characteristics, this review will focus on possible strategies for chemical or physical modification aimed to tailor the final properties of interest.

## 1. Introduction

By the early 1950s, an impetus to learn more about marine organisms arose. The earliest biologically active substance of marine origin was a toxin named holothurin, which was extracted by Nigrelli from a marine organism, the *Actinopyga agassizi* [1]. Holothurin showed some antitumor activities in mice. Since then, the search for drugs and natural products of interest from marine organisms has continued.

The field of natural polysaccharides of marine origin is already large and expanding. Seaweeds are the most abundant source of polysaccharides, as alginates, agar and agarose as well as carrageenans. Table 1 gives an idea of the significant market of these polymers. Even cellulose and amylose have been extracted from the macroalga *ULVA*, which is present along the coasts of Mediterranean Sea and in many lagoons including that of Venice [2]. Chitin and chitosan are derived from the exoskeleton of marine crustaceans.

Recently, microalgae have become particularly interesting because of the possibility to easily control the growth conditions in a bioreactor together with the demonstrated biochemical diversity of these organisms. Greater screening and selection efforts for biologically active compounds, including polysaccharides, have been developed [4]. Examples of microalgae with commercial value are the unicellular red algae *Porphyridium cruemtum* and *P. aerugineum*, because of the large quantities of extracellular polysaccharides they produce [5]. Lewis [6] screened a number of *Chlamidomonas* spp. for extracellular polysaccharide production. The most useful of these is *C. mexicana*, which yields up to 25% of its total organic production as polysaccharides. Moore and Tischer [7] have also reported high extracellular production levels for a number of green and blue-green algae. A number of patents have been issued concerning the production methods and applications for the *Porphyridium* polysaccharide [8]. The *Porphyridium* polysaccharide could also replace existing polysaccharide polymers such as carrageenan in biomedical applications.

Interest is particularly growing towards extreme marine environments. It is obvious that the various extreme marine habitats (deep-sea hydrothermal vents, cold seeps, coastal hot springs, polar regions, hypersaline ponds, *etc.*) should represent a huge source of unknown and uncultivated bacteria. Many microbial exopolysaccharides (EPSs) produced by such extreme bacteria have unique properties; the bacteria must adopt special metabolic pathways to survive in extreme conditions, and so have better capacity to produce special bioactive compounds, including EPSs, than any other microorganisms. Moreover, many thermophylic and hyperthermophylic bacteria can produce EPS under laboratory conditions.

The present review focuses on progress in discovering and producing new marine polysaccharides of interest in pharmaceuticals. The more innovative and appealing fields of application and strategies for their modification are reported. Finally, an updating of recent literature on the more common marine polysaccharides is reported.

## 2. Production, Applications and Modification Strategies of Marine Polysaccharides

### 2.1. Biotechnology of Marine Extremophylic Bacteria

The current opinion of most of the scientific community throughout the world is that knowledge of biochemical processes that adapted in extreme marine environments is the basis for discoveries in biotechnology. The fields of biotechnology that could benefit from miming the extremophiles are very broad and cover the search for new bioactive compounds for industrial, agricultural, environmental, pharmaceutical and medical uses. However, this potential remains to a large extent unexplored and, in respect of the drugs available on the market, only 30% have been developed from natural products and so far less than 10% have been isolated from marine organisms.

Also, if difficulties in culturing marine organisms in the laboratory hold still true today [9,10], the process of industrialization of the microbial products is under recent exploitation. Marine microorganisms are now considered as efficient producers of biologically active and/or chemically novel compounds, and no “supply issue” will appear since scaled-up productions can normally be achieved through bioreactors of any capacity that can be designed nowadays [11,12]. Investigations in shake flasks are conducted with the prospect of large-scale processing in reactors.

Different bioprocess engineering approaches are used for the production of polysaccharides from microorganisms. The major modes of operation in laboratory bioreactors and pilot implants are batch, fed-batch and continuous. Batch growth refers to culturing in a vessel with an initial charge of medium that is not altered by further nutrient addition or removal. This form of cultivation is simple and widely used both in the laboratory and industrially. Growth, product formation and substrate utilization terminate after a certain time interval. Submerged processes, where the organism is grown in a liquid medium, immobilized systems, in which the producing microorganism is restricted in a fixed space, and solid-state processes cultivations, in which the bioprocess is operated at low moisture levels or water activities, are widely employed in batch mode. In a continuous process, fresh nutrient medium is continually added to a well-stirred culture and products and cells are simultaneously withdrawn. Growth and product formation can be maintained for prolonged periods, and the system usually reaches a steady state after a certain period of time. Continuous processes have been used with suspended cells as well as with immobilized cells. In fed-batch culture, nutrients are continuously or semi-continuously fed, while effluent is removed discontinuously. This type of operation is intermediate between batch and continuous processes, increasing the duration of batch cultivation and the overall reactor productivity. The fed-batch process is also applied in several bioprocesses.

### 2.2. Hydrogels and Superporous Hydrogels

Hydrogels based on cross-linked polysaccharides are used in key applications, such as drug delivery systems and tissue engineering. Polysaccharides may also form superabsorbent/superporous hydrogels. Superabsorbent hydrogels are hydrogels having a swelling ratio of a few hundred, and superporous hydrogels are furthermore characterized by interconnected pores with diameters on the micron to millimeter scale. Due to the presence of such big and interconnected pores, superporous hydrogels absorb a considerable amount of water in a very short period of time. These novel products may find applications in the development of drug and protein delivery systems, fast-dissolving tablets, occlusion devices for aneurysm treatment, scaffolding, cell culture, tissue engineering, hygiene products and many others [13].

Sodium alginate, chitosan, agar and carragenaan, in combination with polyacrylics such as poly(acrylic acid) and/or poly(acrylamide), form interpenetrating networks that give rise to superabsorbent and superporous hydrogels of enhanced elasticity [14–16]. Hybrid hydrogels are multi-functional, as their properties depend on cross-linking density and medium pH, and their potential for controlled release is under investigation [17]. Alternatively, polysaccharides have been cross-linked by diacrylates also leading to superabsorbent and/or superporous full-polysaccharide hydrogels [18].

### 2.3. Bioadhesivs and Mucoadhesives from Marine Sources

Natural bioadhesives are polymeric materials that may consist of a variety of substances, but proteins and polysaccharides feature prominently. Many actives can be released via bioadhesives, as steroids, anti-inflammatory agents, pH sensitive peptides and small proteins such as insulin, and local treatments to alleviate pain in the buccal cavity. Requirements for a successful bioadhesive device for topical administration of active agents for prolonged periods of time are:

- maintain intimate contact with the site of application for 1 to 24 hours;
- be sufficiently adhesive and cohesive;
- guarantee controlled delivery of the active ingredients in wet and moist environments;
- be non-toxic, non irritating;
- be easily removable.

Mucosal membranes are lined by epithelial or endothelial cells having “tight junctions” (physiologically connect the enterocytes apically). Membranes are located in or on the skin, ear, eye, nose, gastrointestinal tract. Mucosa have limited permeability to therapeutic agents, especially if the molecular weight is higher than 500 Daltons, thus including most peptides and proteins.

The tenacity with which marine algae cling to ships’ hulls and underwater constructions suggests a remarkable water-resistant adhesive capability. Responsible for fouling growths that reduce efficiency and cause costly damage, they are highly resistant to mechanical removal and all but the most environmentally unacceptable chemical preventive agents. The responsible bioadhesives have extraordinarily high cohesive strength and binding strength to the solid surfaces, enabling the organisms to remain attached under tensional conditions that are, as a matter of fact, comparable to those found in a surgical environment. These qualities have indicated that this is a promising avenue of research in the hunt for more effective tissue adhesives for medical use, for example, surgery closures and bone glue, to replace painful traditional wound closure methods in the first case, and the use of metallic screws in the last one. Various algal bioadhesives have been isolated and characterized to find a safe and efficient candidate to be tested for use on human tissues. They are essentially based on gluing proteins; new formulations comprising polyphenolic proteins from mussels and marine origin polysaccharides are promising, especially as adhesive in ophthalmic therapies.

A bioadhesive system based exclusively on polysaccharides and potentially useful for bone glue has been recently proposed by Hoffmann *et al.* [19]. The authors developed a two-component system based on chitosan and oxidized dextran or starch. The bonding mechanism employs the reaction of aldehyde groups with amino groups in the presence of water, which covalently bind to each other in a Schiff’s base reaction. Chitosan was chosen as amino carrier, and was previously partially depolymerized with acid treatment to obtain a higher ratio between amino and aminoacetyl groups. Aldehyde groups on starch or dextran are generated by oxidation with periodates. In addition, L-DOPA, an important element of mussel adhesives [20–22], was first conjugated to oxidized dextran or starch in analogy to the gluing mechanism of mussels and then oxidized to quinone. The quinone structure of L-DOPA, which is covalently bound to the aldehydes on dextran/starch, can also react with amino groups of chitosan by an imine formation or a Michael adduct formation. All of these reactions result in a strong adhesive force within the glue. With respect to fibrin glue and cyanoacrylate adhesives, which are currently used in clinical practice, biomechanical studies revealed that the new glue is superior to fibrin glue, but has less adhesive strength than cyanoacrylates. Nonetheless, cyanoacrylates, besides having toxic side effects [23], are not resorbable and thus inhibit endogenous bone repair. In conclusion, because both components are natural, biodegradable polysaccharides, without any cytotoxic effects, this bioadhesive seems to be a good candidate for bone or soft tissue gluing applications in surgery.

### 2.4. General Strategies of Modification of Marine Polysaccharides

Natural polysaccharides can play a relevant role in biomedical and pharmaceutical applications, particularly in the field of drug delivery, for their intrinsic biocompatibility and potential low cost. Nevertheless, the properties of such materials sometimes do not fulfill the requirements for specific applications; hence, the development of strategies aiming to chemically and/or physically modify their structure and, consequently, their physical–chemical properties is gaining increasing interest [24,25].

#### 2.4.1. Blending

The technique of blending is particularly attractive as it allows tailoring the properties of interest of the final material in a controlled manner while using polymers already known and widely accepted in the pharmaceutical field. Properties such as biodegradation rate, adhesion to biological substrates, drug solubility inside the polymer matrix, can be modified and tailored to specific applications by simple blending. Further improvements of polymer blends are obtained through (a) addition of a third component, usually a block or graft copolymer, to impart compatibility between the two polymers; (b) chemical modification of one component aimed to create specific interactions with the other one. These strategies can solve problems arising from a bad interaction between the two polymers.

Biodegradable polymer blends usually consist in mixing natural and synthetic biodegradable polymers. Most of polysaccharides are polydispersed in terms of the molecular mass, so they are more similar to synthetic polymers than to biopolymers such as proteins and nucleic acids. Blends based on polysaccharides with natural and synthetic polymers are reported [26]. Blends of alginate with polyvinyl alcohol, a synthetic polymer that has good susceptibility to biodegradation, are proposed due to their good compatibility [27,28]. Addition of glycerol as a natural plasticizer to improve mechanical performances of alginate-based biofilms is also of interest [29].

Blends of different polysaccharides present the advantage that the components are highly compatible, and very homogeneous materials are obtained. Interesting results were obtained for alginate/chitosan blends in which chitosan was previously modified by reaction of part of the amine functionalities with succinic anhydride in order to impart solubility at neutral pH, making it possible to prepare blends of the two polymers from water solution [30,31]. Such blends show a synergistic effect of the chitosan in chelating calcium ions during the alginate gelation process, which in turn results in improved mechanical properties of the corresponding hydrogels. Materials based on such alginate/chitosan blends containing calcium sulfate as osteoinductive phase are promising for applications in bone regeneration [32].

Blends with agar are of interest due to its ability to form reversible gels simply by cooling hot aqueous solutions. This gel-forming property makes blends of agar with biocompatible polysaccharides or synthetic polymers very appealing. Blends with agar usually improve the gelation properties and water-holding capacity of the other polysaccharide component, and the obtained gels are not as strong and brittle as pure agar gels. These characteristics widen the field of potential interest of agar in biomedical applications. Blends of agar with alginate give films that are more flexible and easy to manage than pure agar, and moreover allow modulation of water permeability as a function of the blend composition, and have been proposed for dehydration of fresh fruits [33].

#### 2.4.2. Chemical Modifications

Novel materials based on polysaccharides are being intensively sought, both through bulk and surface modifications. The chemical modification of chitin to produce chitosan represents the most fundamental process in this essay. This chemical modification is in fact particularly simple, since it just involves the hydrolysis of an amide moiety to generate the corresponding primary amino function. As in all polymer modifications, the ideal 100% conversion is very hard to achieve, and chitosans are therefore a whole family of polymers, characterized by their average molecular weight and their degree of deacetylation, *i.e.*, the percentage of amide groups converted into NH2 counterparts.

The ubiquitous hydroxyl groups in polysaccharides are the most obvious source of chemical modification that has been exploited, although all other functionalities present on polysaccharides (amino, acid, carboxylate) have been used for chemical reactions. A variety of chemical modifications (theoretically, all the reactions involving these functional groups may be performed on polysaccharides) have been realized. Hydroxyls are used for oxidation reactions with peroxide to generate the more reactive aldheyde groups, for esterification with acid or anhydrides to reduce hydrophylicity, for sulfonation reactions with a variety of sulfonating agents, for bromination or chlorination. Amino groups are more reactive than hydroxyls, so they must be protected to selectively address the reaction onto hydroxyls. Amines are specifically used for aqueous carbodiimide chemistry. Modified polysaccharides are used as such or employed for successive reactions, including copolymerization. Several examples of the more explored chemical reactions that involve hydroxyls as well as other groups on polysaccharides, widely employed to modify properties such as solubility, to impart novel characteristics to the plain polymer, or to create sites for specific binding or interactions with other molecules of biological interest, are hereafter reported.

##### 2.4.2.1. Hydrophobic Modification

Hydrophilic polymers modified by hydrophobic moieties represent a combination of surfactant and polymer properties in one molecule. As a consequence, they self-associate in aqueous solution to form complex micellar structures. This feature can be of interest for medical diagnosis application, as micelles are used as coating of colloidal metal particles for labeling biomolecules in immunological assays [34].

Hydrophobically-modified polysaccharides are polysaccharides partially modified by cholesterol or other hydrophobic moieties. They are used as coating to stabilize liposomes for chemotherapy and immunotherapy [35]. A selective uptake by cancer cells, particularly by human colon and lung cancer cells, has been shown by liposomes coated with cholesterol derivatives of polysaccharides bearing 1-aminolactose [36]. Different cholesterol-linked celluloses of various origins, intended for applications as bile acid/cholesterol sequestrants, were prepared by reaction with monocholesteryl succinate [37]. The authors found that the introduction of a mesogenic substituent may induce a thermotropic behavior with formation of a mesophase. It has been also supposed that thermotropic cholesterol derivatives of polysaccharides would have even enhanced bile acid/cholesterol sequestration, probably through mixed mesophase formation with the free bile acids/cholesterol.

##### 2.4.2.2. Depolymerization

Depolymerization is often used to reduce molecular weight in view of specific applications, overall as patches for controlled release of drugs, because low molecular weight polymers have an increased amount of polar end groups. The patches are adhesives based on chemically and physically modified polysaccharides, which are partially depolymerized to provide a more effective topical and transdermal drug delivery system. Such patches were really found to be highly adhesive while providing superior drug penetration.

Classical depolymerization methods include ozonolysis in aqueous solutions [38], oxidative depolymerization induced by oxygen radical generating systems [39,40], specific enzymatic degradation [41]. A recent patent reports on a method for carrying out a targeted depolymerization of polysaccharides at increased temperatures, producing simultaneously polysaccharide derivatives with a desired degree of polymerization [42]. The molecular weight of polysaccharides can be also reduced through chemical hydrolysis, ionizing radiation or electronic beam radiation [43–45].

##### 2.4.2.3. Sulfation

Sulfated polysaccharides are polysaccharides containing high amounts of sulfate groups. They can be found in nature, but a lot of sulfated polysaccharides have been obtained by chemical modification. As sulfonating agents, sulfur trioxide-pyridin, piperidine-*N*-sulfonic acid, sodium sulfite and chlorosulfonic acid are the most used. The reaction may involve all the hydroxyls (primary and secondary) and the amines eventually present on polysaccharide, or it can be targeted to a specific site. Sulfated polysaccharides are known to have anti-retroviral [46] and/or antimalarial activity [47]. This last can further be enhanced by a combination with artemisinin or its di-hydro derivative [48]. Sulfated chitin and chitosan are found to be efficient carriers to deliver therapeutic agents across a mucosal membrane [49].

**Figure 1:**
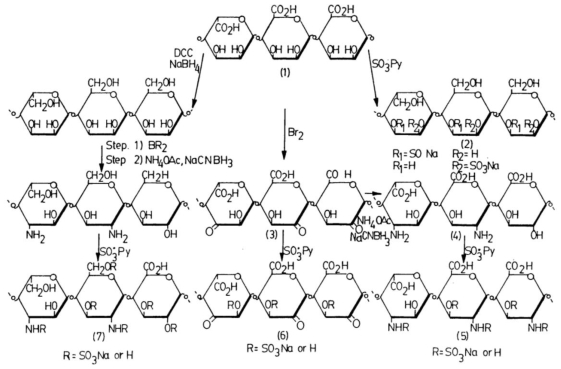
The synthetic strategy for preparing alginic acid sulfated derivatives.

**Figure 2:**
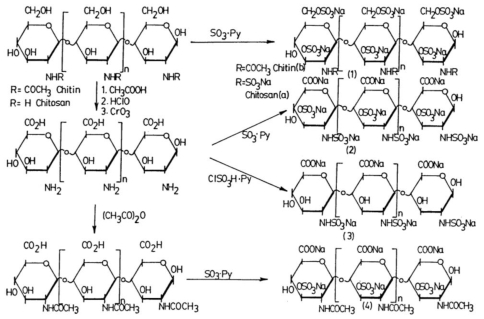
The synthetic strategy for preparing chitin and chitosan sulfated derivatives [50].

A method for inhibiting or decreasing intestinal cholesterol and fatty acids absorption in man by oral administration of synthetic sulfated polysaccharides has been the matter of an American Patent [50]. The invention is based upon the discovery that sulfated polysaccharides are potent inhibitors of human pancreatic cholesterol esterase, the enzyme responsible for promoting the intestinal absorption of cholesterol and fatty acids. A variety of polysaccharide polymers can be sulfated to produce potent inhibitors of human pancreatic cholesterol esterase. Increasing inhibitory activities are realized from increased molecular weights and sulfation at a specific position; increased efficacy is obtained by reducing absorption of the polysaccharides. Accordingly, the methodology includes non-absorbable sulfated polysaccharides having a molecular weight greater than 10 kDa; furthermore, the presence of a 3-sulfate on the sugar ring markedly enhances inhibition. Alginic acid, chitin and chitosan, agar, as well as other abundant and cheap natural polysaccharides as pectin (from vegetables and fruits), dextran and cellulose (from plants and trees), have been reacted in a controlled manner to produce sulfated derivatives. These derivatives are all water soluble, potent inhibitors of human pancreatic cholesterol esterase, whereas the parent starting polymers are either not inhibitory or poorly inhibitory. These sulfated polysaccharides can be administrated orally in pharmaceutical forms such as tablets, capsules, liquids and powders. Sulfation of polysaccharides was obtained by reaction with sulfur trioxide-pyridin. In the case of alginic acid, the reaction was performed directly on native polymer, or on the oxidized polymer, or on the product of oxidation followed by reductive amination of the native alginic acid, leading to different levels of sulfation (Figure 1). For chitin and chitosan, it is possible to target the reaction on two sites or on only one specific site, following different routes of synthesis (Figure 2).

## 3. Examples of Applications of More Abundant Marine Polysaccharides in Pharmaceuticals

### 3.1. Alginate

Alginate is a natural occurring polysaccharide of guluronic (G) and mannuronic (M) acid, quite abundant in nature as structural component in marine brown algae (*Phaeophyceae*) and as capsular polysaccharides in soil bacteria. Brown algal biomass generally consists of mineral or inorganic components and organic components, these last mainly composed by alginates, fucans, and other carbohydrates. The isolating process of alginates from brown algal biomass is simple, including stages of pre-extraction with hydrochloric acid, followed by washing, filtration, and neutralization with alkali. Sodium alginate is precipitated from the solution by alcohol (isopropanol or ethanol) and usually re-precipitated (to achieve higher purity) in the same way. However, the real processing scheme for alginate production is quite complicated, including 15 steps [51].

Alginate instantaneously forms gel-spheres at pH > 6 by ionotropic gelation with divalent cations such as Ca2+ [52], Ba2+, or Zn2+ and for this it is widely used for microencapsulation of drugs. On the other hand, at low pH, hydration of alginic acid leads to the formation of a high-viscosity “acid gel”. The ability of alginate to form two types of gel dependent on pH, *i.e.*, an acid gel and an ionotropic gel, gives the polymer unique properties compared to neutral macromolecules, and it can be tailor-made for a number of applications.

The microencapsulation technique has been developed particularly for the oral delivery of proteins, as they are quickly denaturated and degraded in the hostile environment of the stomach. The protein is encapsulated in a core material that, in turn, is coated with a biocompatible, semi permeable membrane, which controls the release rate of the protein while protecting it from biodegradation. Due to its mild gelation conditions at neutral pH, alginate gel can act as core material in this application, while poly(ethylene glycol) (PEG), which exhibits properties such as protein resistance, low toxicity and immunogenicity [53], together with the ability of preserving the biological properties of proteins [54,55], can act as a coating membrane. A chitosan/PEG-alginate microencapsulation process [56], applied to biological macromolecules such as albumin or hirudin [57], was reported to be a good candidate for oral delivery of bioactive peptides.

Several examples of alginate-encapsulated drugs, other than proteins, can also be found in literature. Qurrat-ul-Ain *et al.* [58] reported that alginate microparticles showed better drug bioavailability and reduction of systemic side effects compared with free drugs in the treatment of tuberculosis. Polyelectrolyte coating of alginate microspheres showed to be a promising tool to achieve release systems characterized by approximately zero-order release kinetics, release up to 100% of entrapped drug (dexamethasone) within 1 month, and improved biocompatibility [59].

Composites technology has been applied to alginate for drug delivery purposes. As an example, montmorillonite-alginate nanocomposites have been recently proposed as a system for sustained release of B1 and B6 vitamines [60]. The vitamins intercalated in the nanocrystals of the inorganic phase, and successively the hybrid B1/B6 montmorillonite (MMT) is further used for the synthesis of B1/B6-MMT-alginate nanocomposite.

In their simplest design, oral controlled-release dosage forms made from alginates are monolithic tablets in which the drug is homogeneously dispersed. Drug release is controlled by the formation of a hydrated viscous layer around the tablet, which acts as a diffusional barrier to drug diffusion and water penetration. Water soluble drugs are mainly released by diffusion of dissolved drug molecules across the gel layer, while poorly soluble drugs are mainly released by erosion mechanisms. Modulation of drug release rate has been achieved by incorporating pH-independent hydrocolloids gelling agents or adding polycationic hydrocolloids such as chitosan [61,62]. A number of mucoadhesive systems based on alginate have been developed [63,64]. The main shortcoming of alginates consists in their rapid erosion at neutral pH; furthermore, the adhesion to mucosal tissues is reduced when cross-linked with divalent cations. Alginates have been extensively used to modify the performances of other polysaccharides, such as chitosan, through the realization of alginate coated chitosan microspheres [65]. In the literature, it is also possible to find acrylic modified polysaccharides developed with the aim to obtain a finer control over release rate or to improve adhesive properties [66,67].

The modification of sodium alginate with amine and/or acid moieties with the aim to optimize their properties for drug delivery applications by modulating the time of erosion, the rate of release of drugs, and the adhesion to substrates has been attempted by Laurienzo *et al*. [68]. In this article, graft copolymers based on alginate and acrylic polymers were synthesized by radical polymerization of the acrylic monomers or oligomers in the presence of sodium alginate. The authors found that the modification of alginate network significantly affects water uptake and erosion rate of matrices prepared by direct compression. As a consequence, the release rate and mechanism of a highly soluble-low molecular weight drug was found to be controlled mainly by either a diffusion/erosion or erosion mechanism, depending on polymer type and medium pH. Furthermore, they found increased adhesive properties of the copolymers, and concluded that they might be good candidates in formulations for mucoadhesive systems for the treatment of heartburn and acid reflux, because of the possibility to improve the gastric protective coating action.

Hydrophobically-modified alginates can be prepared by oxidation followed by reductive amination of the 2,3-dialdehydic alginate [69]. The ability of alginate to bind cations renders these modified alginates interesting as biosurfactants environmentally friendly for the removal of organics and metal divalent cations.

Among the possible applications of alginate gel systems, one of the most promising is for tissue regeneration. The main drawback of alginate matrix gels is represented by their high density of network, which limits the cell growth [70]; moreover, cell anchorage, a strict requirement for proliferation and tissue formation, is limited on alginate gels, because of its hydrophilic nature. PEG copolymers are used to improve the biocompatibility of polysaccharides. Several PEG-alginate systems for cell entrapment have been reported [71]; recently, a new alginate-g-PEG copolymer has been described [72]. The synthesis goes via hydrophobization of alginate with alkylic amines; successively, the secondary amines grafted onto the alginate react with a low molecular weight PEG functionalized with a carboxylic acid group at one end, to produce an alginate-g-PEG copolymer. As the reactions do not involve the carboxylic acid groups of the alginate, the ability to cross-link via ionic interactions is retained. The obtained copolymers show the double function to form micelles in water above a critical concentration and to form gels with divalent cations.

#### Alginate for Wound Healing

Alginate dressings for wound healing have been successfully applied for many years to cleanse a wide variety of secreting lesions, and they still remain widely used in many circumstances. Alginate gels dressings are highly absorbent, and this limits wound secretions and minimizes bacterial contamination. Alginate fibers trapped in a wound are readily biodegraded [73]. Alginate dressings maintain a physiologically moist microenvironment that promotes healing and the formation of granulation tissue. Alginates can be rinsed away with saline irrigation, so removal of the dressing does not interfere with healing granulation tissue. This makes dressing changes virtually painless. Alginate dressings are very useful for moderate to heavily exudating wounds [74].

The healing of cutaneous ulcers requires the development of a vascularized granular tissue bed, the filling of large tissue defects by dermal regeneration, and the restoration of a continuous epidermal keratocyte layer. These processes were modeled *in vitro* in one study utilizing human dermal fibroblasts, microvascular endothelial cells (HMEC), and keratocyte cultures [75]. In this study, the calcium alginate was found to increase the proliferation of fibroblasts but decreased the proliferation of HMEC and keratocytes. In contrast, the calcium alginate decreased fibroblast motility but had no effect on keratinocyte motility. There was no significant effect of calcium alginate on the formation of capillary-like structures by HMEC. The effects of calcium alginate on cell proliferation and migration may have been mediated by released calcium ions. These results suggest that the calcium alginate tested may improve some cellular aspects, but not others.

Alginates have been shown to be useful also as hemostatic agents for cavity wounds [76]. A study compared the effects of calcium and zinc containing alginates and non-alginate dressings on blood coagulation and platelet activation [77]. The study showed that alginate materials activated coagulation more than non-alginate materials. The extent of coagulation activation was affected differently by the alginate M or G group concentration. Moreover, it was demonstrated that alginates containing zinc ions had the greatest potentiating effect on prothrombotic coagulation and platelet activation. However, there has been one report of a florid foreign body reaction after the use of an alginate dressing to obtain hemostasis in an apicectomy cavity. The case suggests that alginate fibers left *in situ* may elicit a long-lasting and symptomatic adverse foreign body reaction [78].

### 3.2. Chitosan

Chitosan is a copolymer of β-(1→4)-linked 2-acetamido-2-deoxy-D-glucopyranose and 2-amino-2-deoxy-D-glucopyranose. It is obtained by deacethylation of the natural occurring chitin. Chitin is extracted from the exoskeleton of marine organisms, mainly crabs and shrimps, as described by Burrows [79]. Briefly, the exoskeletons are crushed and washed, then treated with boiling sodium hydroxide to dissolve the proteins and sugars, thus isolating the crude chitin. The major applications of chitosan are in biomaterials, pharmaceuticals, cosmetics, metal ion sequestration, agriculture, and foodstuff treatment (flocculation, clarification, *etc.*, because of its efficient interaction with other polyelectrolytes). Development of chitosan chemistry is relevant in biomedical science, particularly in the topic of drug delivery [80,81]. Unlike its precursor chitin, which is insoluble in most common solvents, chitosan can readily be spun into fibers, cast into films, or precipitated in a variety of micromorphologies from its acidic aqueous solutions. Electrospinning from acetic acid solutions to provide nanofibers has also been reported [82]. The excellent ability to form porous structures simply by freezing and lyophilizing its solutions, or by simple techniques such as “internal bubble process” [83], makes chitosan a versatile biopolymer for tissue engineering, particularly in orthopedics for cartilage [84] and bone regeneration [85]. Possible chitosan matrix preparations for cell cultures include gels [86], sponges [87], fibers [88], or porous compositions of chitosan with ceramic [89] or other polymeric materials such as collagen or gelatin [90,91]. Chitosan has been combined with a variety of materials such as alginate, hydroxyapatite, hyaluronic acid, calcium phosphate, PMMA, poly-L-lactic acid (PLLA), and growth factors for potential application in cell-based tissue engineering [92]. Alginate is a candidate biomaterial for cartilage engineering but exhibits weak cell adherence. Iwasaki *et al*. [93] reported on alginate-based chitosan hybrid polymer fibers which showed increased cell attachment and proliferation *in vitro* compared to alginate fibers. In addition, when it is combined with alginates the system acts as a biomimetic membrane which controls the release of bioactive macromolecules such as hirudin [94]. Interpenetrated polymer networks (IPNs) of chitosan and poly(acrylic acid) (PAA) for applications as biodegradable filling systems and controlled release devices have been prepared by radical polymerization of acrylic acid in the presence of chitosan [95].

In orthopedics, chitosan is used as an adjuvant with bone cements to increase their injectability while keeping the chemical-physical properties (time setting and mechanical characteristics) suitable for surgical use [96].

Chitosan can be modified in order to improve compatibility in blends with other polymers and to impart solubility in water or in common organic solvents such as chloroform, pyridine, tetrahydrofuran. Organically soluble derivates of chitosan can be used to formulate by-designed materials for biomedical applications such as polymeric drugs and artificial organs. Acylation, alkylation and phthaloylation reactions have been widely used [97–101]. Novel formulations based on a combination of chitosan with polyol-phosphate salts have allowed neutral solutions to be obtained without any chemical modification of the chitosan [102]. These formulations possess a physiological pH and can be held liquid below room temperature for encapsulating living cells and therapeutic proteins; they form monolithic gels at body temperature. Therefore, when injected *in vivo* the liquid formulations turn into gel implants *in situ*. This system was used successfully to deliver biologically active growth factors *in vivo* as well as an encapsulating matrix for living chondrocytes for tissue engineering applications.

Due to its favorable gelling properties, chitosan can deliver morphogenic factors and pharmaceutical agents in a controlled fashion. Methodologies to produce micro and nanoparticles of chitosan and its derivates for drug delivery include spray-drying and water-in-oil emulsions techniques. Drug release from chitosan microparticles can be controlled by cross-linking the matrix. Microparticles can be cross-linked by chemicals such as glutaraldheyde, genipin, diisocyanates and ethylene glycol diglycidyl ether, or by ionic agents, or by a combination of them [103]. Among chemical agents, genipin, a naturally occurring cross-linker, significantly less cytotoxic than glutaraldehyde [104], has been largely employed for crosslinking of hydrogels and beads. Among ionic agents, tri-polyphosphate (TPP), a non toxic and multivalent polyanion, was reported to form gel by ionic interaction between positively charged amino groups of chitosan and negatively charged counterion of TPP [105–108]. By controlling the pH and the concentration of TPP solutions, cross-linked chitosan microspheres for a potentially controlled release of drugs have been obtained [109]. In alginate-chitosan mixed systems, cross-linked microcapsules are prepared either by dropping a solution of sodium alginate into an acidic solution containing chitosan, or by incubating calcium alginate beads in a chitosan solution, or by dropping a chitosan solution into a TPP solution containing sodium alginate [110].

Many applications have been proposed for chitosan-based delivery devices. Strong electrostatic interactions with mucus open the doors to possible applications as gastrointestinal or nasal delivery systems [111,112]. Additionally, the cationic character of chitosan imparts particular possibilities. pH-sensitive microspheres made of hybrid gelatine/chitosan polymer networks have been developed and showed to release drugs only in acidic medium [113]. A pH-sensitive, chitosan-based hydrogel system for controlled release of protein drugs cross-linked by genipin was developed also by Chen *et al.* [114].

Brush-like copolymers of chitosan with poly(ɛ-caprolactone) (PCL), polyethylene glycol (PEG) or their copolymers have been prepared following different procedures of synthesis. [115–119]. Such amphiphilic copolymers are able to self-assembly in aqueous solutions. Nanomicelles obtained from chitosan-*g*-PCL copolymers have been successfully tested as carriers of hydrophobic drugs [120].

Chitosan and its derivates or salts have been extensively investigated for alternative routes of administration of insulin, such as oral, nasal, transdermal and buccal delivery systems [121,122]. Chitosan is mucoadhesive and able to protect the insulin from enzymatic degradation, prolong the retention time of insulin, as well as open the inter-epithelial tight junction to facilitate systemic insulin transport. Targeted delivery of insulin is deemed possible in future through using chitosan with specific adhesiveness to the intended absorption mucosa. Water-soluble low molecular weight chitosan renders insulin able to be processed under mild conditions, and sulfated chitosan markedly opens the paracellular channels for insulin transport. The development of insulin carriers using chitosan base and/or its derivates involves many techniques such as nanocomplexation, mixing, solubilization, ionotropic gelation, coacervation, layer-by-layer encapsulation, water-in-oil emulsification, membrane emulsification, lyophilization, compression, capsulation, casting, adsorption and/or absorption processes.

The cationic nature of chitosan allows it to complex DNA molecules, making it an ideal candidate for gene delivery strategies. Chitosan nanoparticles appear to control the release of DNA and prolong its action both *in vitro* and *in vivo* [123–125]. Chitosan in fact provides protection against DNAase degradation [126]. Chitosan was selected also as a coating material, alone or in blends with polyvinylalcohol, for cationic surface modification of biodegradable nanoparticles for gene delivery [127]. These cationic surface modified nanospheres can readily bind DNA by electrostatic interaction, simply mixing their aqueous solutions.

Chitosan nanoparticles have been used also to transport small interfering RNAs (siRNA). The cellular RNA interference machinery is now used in cancer gene therapy to turn off, for instance, oncogene expression. Katas and Alpar [128] first studied the interaction behavior of siRNA and chitosans given that the structure and size of siRNA are quite different to that of pDNA. Studies *in vitro* demonstrated that chitosan nanoparticles are able to mediate gene silencing. Furthermore, the transfection efficiency of RNA depends on its association with chitosan. Indeed, entrapping siRNA using ionic gelation showed a better biological effect than simple complexation or siRNA adsorption onto the chitosan nanoparticles. This might be attributed to stronger interactions between the chitosan and siRNA and a better loading efficiency when using ionic gelation.

Another interesting property of chitosan is its intrinsic antibacterial activity [129]. For this reason, chitosan is a preferred carrier for drug delivery of antibiotics, combining its intrinsic antibacterial activity with that of the bound antibiotic. Studies have shown that chitosan may reduce the rate of infections induced by bacteria such as *Staphylococcus* in rabbits [130]. The mechanism involves its cationic amino groups, which associate with anions on the bacterial cell wall, suppressing biosynthesis; moreover, chitosan disrupts the mass transport across the cell wall accelerating the death of bacteria. The antibacterial activity is retained also when it is combined with other polymers [131]. Blends of chitosan with polyvinyl alcohol (PVA) crosslinked by gamma radiation have been prepared for wound dressing [132]. Chitosan was used to prevent microbiological growth, such as fungi and bacteria, on the PVA polymer. Moreover, chitosan has antiacid and antiulcer characteristics, which prevents or weakens drug irritation in the stomach [133].

Nanostructured surface coatings based on polysaccharides have been obtained by a combination of chitosan with hyaluronic acid and heparin [134]. The engineering of the nanoscale surface features of biologically active materials is an important goal for biomaterials scientists, as it strongly influences a variety of responses of mammalian cells towards biomaterials.

### 3.3. Agar/Agarose and Carrageenans

Agar (or agar-agar) is a phycocolloid, which is constructed from complex saccharide molecules (mainly, β-D-galactopyranose and 3,6-anhydro-α-L-galactopyranose units) extracted from certain species of red algae (*Gelidium*, *Gelidiela*, *Pterocladia*, *Gracilaria*, *Graciliaropsis*, and *Ahfeltia*) [135]. Genetic manipulation of agarophytes in the development stages promises to minimize seasonal variations in plant growth and agar quality. Agar and its variant agarose contain also variable amounts of sulfate, piruvate and uronate substituents.

Agar is insoluble in cold water but is soluble in boiling water. Agar dissolved in hot water and permitted to cool will form thermally reversible gels (the gel will melt when heated and reformed again when cooled), without the need of acidic conditions or oxidizing agents. This characteristic gives agars the ability to perform a reversible gelling process without losing their mechanical and thermal properties. The significant thermal hysteresis of the gel is another important property for commercial applications. The gelling process in agar is due to the formation of hydrogen bonds in a continuous way [136–139]. Gelation occurs as a result of a coil-double helix transition [140–142]; helices interact among themselves and the gel is formed by linked bundles of associated right-handed double helices. The resulting three-dimensional network is capable of immobilizing water molecules in its interstices [143]. Characteristically, solutions containing 1–2% agar by weight will gel at about 35 °C and will melt at about 85 °C. The 1–2% (w/w) agar gels are strong and brittle. Typically, a force of 500–1000 g cm^−2^ is required to break these gels. The strength and brittleness of agar gels are proportional to the amount of 3-6-anhydro-α-L-galactopyranose in the agar. An alternatively way to cross-link agar is via chemical agents [144]. By crosslinking with glutaraldehyde, agar forms superabsorbent hydrogels.

The ionic nature of the agar molecules permits to complex with proteins. The presence of proteins in wine, juice and vinegar clouds these products. Agar is added during processing to bind with proteins impurities. This facilitates the removal of proteins by filtration or centrifugation.

Agar has medicinal or pharmaceutical industrial applications including use as suspending agent for radiological solutions (barium sulfate), as a bulk laxative as it gives a smooth and non-irritating hydrated bulk in the digestive tract, and as a formative ingredient for tablets and capsules to carry and release drugs. Pharmaceutical grade agar has a viscous consistency. In microbiology, agar is the medium of choice for culturing bacteria on solid substrate. Agar is also used in some molecular microbiology techniques to obtain DNA information [145]. More recently, agar was used in a newly-developed medium, *i.e.*, combined deactivators-supplemented agar medium (CDSAM), to evaluate the viability of dermatophytes in skin scales [146]. The experimental data from this clinical study indicate that CDSAM was more useful than standard media in accurately evaluating the efficacy of antifungal drugs. Agar proportion method for drug susceptibility tests has been used since 1957 [147]. Recently, the test has been replaced by more rapid tests [148].

The possibility to use agar and agarose beads for sustained release of water soluble drugs has been investigated [149]. Agarose has a significantly lower sulfate content, better optical clarity and increased gel strength with respect to agar, but it is considerably more expensive [150]. Agar beads containing phenobarbitone sodium as a water soluble and hypnotic drug were prepared [151]. The encapsulation procedure consists in dissolving the drug in a hot (around 70 °C) agar aqueous solution and then dropping the solution in a cold bath containing a non-solvent for agar (acetone or ethyl acetate). Agar beads instantaneously form by gelification. The results of dissolution and release studies indicated that agar beads could be useful for the preparation of sustained release dosage forms, although no many further studies have been developed.

Carrageenan, as well as agar and agarose, is a sulfated polysaccharide obtained by extraction with water or alkaline water of certain *Rhodophyceae* (red seaweed). It is a hydrocolloid consisting mainly of the potassium, sodium, magnesium, and calcium sulfate esters of galactose and 3,6-anhydro-galactose copolymers.

Plain carrageenans, as well as agar, are mainly used as food additive, but increasing attention is given to possible biomedical applications, in combination with synthetic polymers. The synthesis of agar-graft-polyvinylpyrrolidone (PVP) and k-carrageenan-graft-PVP blends by a microwave irradiation method has been reported [152]. The physicochemical and rheological properties of the corresponding hydrogels were studied and compared with control agar and k-carrageenan hydrogels. The novel blend hydrogels were found to be not as strong and showed better spreadability and water-holding capability, so they are potentially useful in moisturizer formulations and active carriers of drugs. The use of blended PVP with agar in hydrogel dressings has also been reported [153].

### 3.4. Exopolysaccharides (EPS)

Microbial polysaccharides represent a class of important products that are of growing interest for many sectors of industry. The advantages of microbial polysaccharides over plants polysaccharides are their novel functions and constant chemical and physical properties. A number of common marine bacteria widely distributed in the oceans can produce EPSs; nevertheless, most of these EPSs remain poorly understood, and only a few of them have been fully characterized. The roles of microbial EPSs in the ocean are briefly described in Table 2.

In recent years, there has been a growing interest in isolating new exopolysaccharides (EPSs)-producing bacteria from marine environments, particularly from various extreme marine environments [154]. Many new marine microbial EPSs with novel chemical compositions, properties and structures have been found to have potential applications in fields such as adhesives, textiles, pharmaceuticals and medicine for anti-cancer, food additives, oil recovery and metal removal in mining and industrial waste treatments, *etc*. General information about the EPSs produced by marine bacteria, including their chemical compositions, properties and structures, together with their potential applications in industry, are widely reported [155]. Components more commonly found in marine EPSs are listed in Table 3. Some more recent and specific examples from literature are hereafter illustrated.

### 3.4.1. Biological Activity of EPSs

EPS2, a polysaccharide produced by a marine filamentous fungus *Keissleriella* sp. YS 4108 exhibited profound free radical-scavenging activities [156,157]. Antioxidants are commonly used in processed foods, as they could alleviate the oxidative damage of a tissue indirectly by increasing cells’ natural defenses and directly by scavenging the free radical species [158,159]. As most of antioxidants used are synthetic and have been suspected of being responsible for liver damage and carcinogenesis [160,161], it is essential to develop and utilize effective natural antioxidants. Sun, Mao *et al.* [162] isolated three different exopolysaccharides from marine fungus *Penicillium* sp. F23-2, and evaluated their antioxidant activity by assays in *in vitro* systems. The results showed that the three polysaccharides possessed good antioxidant properties, especially scavenging abilities on superoxide radicals and hydroxyl radicals.

Interesting studies concern with the roles of carbohydrates as recognition sites on the cell surfaces [163]. It has been demonstrated that micro-organisms must specifically attach to the host cell to avoid being washed away by secretions. Such attachment would permit colonization or infection, sometimes followed by membrane penetration and invasion. Bergey and Stinson [164] and Bellamy *et al*. [165] provide some evidence of the participation of the carbohydrates in the cellular recognition process of the interaction between host and pathogen. It is an attractive possibility to use carbohydrate-based drugs for blocking the early stages of an infection process. In particular, sulfated polysaccharides are involved in biological activities such as cell recognition, cell adhesion, or regulation of receptor functions [166–168]. Obtaining sulfated polysaccharides from algae has made development of biotechnology for obtaining new therapeutic products easier. The use of bioactive compounds from microalgae has been considered recently as an alternative to prevent microbial infections in animals and humans and to decrease the use of antibiotics [169]. Ascencio *et al*. [170] identified heparan sulfate glycosaminoglycan as putative host target for *Helicobacter pylori* adhesion. A number of marine and freshwater microalgal strains for the production of sulfate exopolysaccharides was screened by these authors to evaluate whether these exopolysaccharides can block adherence to human and fish cells of human gastrointestinal pathogens, such as *H. pylori* and *Aeromonas veronii.* Results indicate the sulfated polysaccharides of some species of microalgae inhibit the cytoadhesion process of *H. pylori* to animal cells. The treatment with sulfated polysaccharides could so be used to block the initial process of colonization of the host by *H. pylori*, so it may represents an alternative prophylactic therapy in microbial infections where the process of cytoadhesion of host to pathogen is likely to be blocked. Such a therapy could replace the use of antibiotics and antiparasitic drugs that are always aggressive towards the host and, moreover, can generate resistant strains to the antibiotic, making it necessary to use second generation antibiotics [171,172].

Many polysaccharides have an anticancer activity. In general, the action mechanism is via macrophage activation in the host [173,174]. More recently, Matsuda *et al.* [175] reported that a sulfated exopolysaccharide produced by *Pseudomonas* sp. shows a cytotoxic effect towards human cancer cell lines such as MT-4. These findings have resulted in further interest in this polysaccharide as a new anticancer drug suitable for clinical trials.

Several marine bacteria isolated from deep-sea hydrothermal vents have demonstrated their ability to produce, in aerobic conditions, unusual EPS. These EPS could provide biochemical entities with suitable functions for obtaining new drugs. They present original structural feature that can be modified to design compounds and improve their specificity.

Studies aimed to discover biological activity of such new EPS were performed by Colliec-Jouault *et al*. [176]. An EPS secreted by *Vibrio diabolicus*, a new species isolated from *Alvinella pompejana* of Pompei, was evaluated on the restoration of bone integrity in an experimental model and was demonstrated to be a strong bone-healing material. High molecular weight EPS produced from fermentation of *Vibrio diabolicus* stocks has been implanted in bone injuries created in the cranium of a mouse, and compared with analogous injuries not treated or treated with collagene as a reference. After 15 days, a 95% healing was found for the injuries treated with the EPS, while less than 30% healing was found for untreated or collagene treated injuries.

Another EPS produced by *Alteromonas infernos*, a new *Alteromonas* species isolated in a hydrothermal vent from the Guaymas region, was really modified in order to obtain new heparin-like compounds [177]. The native EPS was depolymerized by radical mechanism or chemical hydrolysis, and low-mass derivatives (around 24,000 Da) were sulfated to obtain an “heparin-like” or “heparin-mimetic” compound. Unlike the native EPS, the resulting sulfated EPS presented anticoagulant properties as heparin.

### 3.4.2. Exopolysaccharides from Cyanobacteria

Cyanobacteria, also known as blue-green algae or blue-green bacteria because of their color (the name comes from Greek: kyanós = blue), are a significant component of the marine nitrogen cycle and an important primary producer in many areas of the ocean. They are also found in habitats other than the marine environment; in particular, cyanobacteria are known to occur in both freshwater, hypersaline inland lakes and in arid areas where they are a major component of biological soil crusts.

Since the early 1950s, more than one hundred cyanobacteria strains of different genera have been investigated regard to the production of exocellular polysaccharides. Such polysaccharides are present as outermost investments forming sheaths, capsules and slimes that protect the bacterial cells from the environment. Moreover, most polysaccharide-producing cyanobacteria release aliquots of capsules and slimes as soluble polymers in the culture medium (RPS)[178]. In recent years the interest towards such cyanobacteria has greatly increased, in particular towards those strains that possess abundant capsules and slimes and so release large amount of soluble polysaccharides, which can be easily recovered from liquid culture.

The chemical and rheological analyses show that RPS are complex anionic hetero-polymers, in about 80% cases containing six to ten different monosaccharides, glucose being the most abundant [179]. This characteristic is unusual in EPS of industrial interest, which usually contain a lower number of monosaccharides, and is of great significance [180]. Actually, a large number of different monosaccharides in only one polymer makes many structures and architectures possible [181], thus increasing the chance of having a polymer with peculiar properties, not common to currently utilized products. The rheological properties of RPSs’ aqueous solutions make them useful as thickening agents for water solutions, together with their ability to stabilize the flow properties of their own solutions under drastic changes of pH, temperature and ionic strength [182,183].

Another important feature of such RPS is their anionic nature. In fact, in about 90% cases, one or more uronic acids are present; moreover, RPS also contain sulfate groups. Both uronic and sulfate groups contribute to impart a high anionic density to the polymer. The anionic charge is an important characteristic for the affinity of these EPS towards cations, notably metal ions. Almost all RPS have significant levels of non-saccharidic components, such as ester-linked acetyl and/or pyruvyl groups and peptidic moieties which, along with other hydrophobic components as the deoxysugars fucose and rhamnose, contribute to a significant hydrophobic behavior of these otherwise hydrophilic macromolecules, conferring them emulsifying properties [184,185].

The presence of charged groups on the macromolecules may lead to several interesting industrial applications: their capability to bind water molecules can be exploited by the cosmetic and pharmaceutical industries for product formulations [186]. A promising new field of application that attracted much attention is related to antiviral activity of some RPSs isolated from the blue-green alga *Spirulina platensis* [187,188]. The presence in these polysaccharides of significantly high amounts of sulfate groups, indeed, is accounted for their antiviral activity, and an increasing amount of data is available.

## 4. Conclusions

A brief review of recent advances in applications of polysaccharides of marine origin in the medical and pharmaceutical fields has been reported. Many experimental results clearly indicate that novel exciting and promising marine sources of polysaccharides of applied interest, such as cyanobacteria and thermophylic or hyperthermophylic bacteria from extreme habitats, are emerging. Further investigations with a multidisciplinary approach are imperative in order to develop novel polymers useful as drugs or for healthcare in a larger sense.

## Figures and Tables

**Table 1:** Polymers from macro-algae: 2003 market data [3].

Product	Production (t y^−1^)	Algae Harvested (t y^−1^)	Comments
Carrageenan	33,000	168,400	Mainly *Eucheuma* and *Kappaphycus*
Alginate	30,000	126,500	*Laminaria*, *Macrocystis*, *Lessonia*, *Ascophyllum* and others
Agar	7,630	55,650	Mainly *Gelidium* and *Gracilaria*

**Table 2:** Some roles of microbial exopolymeric material (EPSs) in the marine environment. Adapted from [151].

Role of Exopolymer	Example
Assists in attachment to surfaces	Exopolymers of marine *Vibrio* MH3 were involved in reversible attachment. Cross-linking of adjacent polysaccharide chains aided in permanent adhesion.
Facilitates biochemical interactions between cells	Exopolymer mediated bacterial attachment to the polar end of blue-green N2-fixing alga. EPS aided attachment to symbiotic host such as vent tube worm to absorb metals and detoxify microenvironment. Exopolymer buffered against sudden osmotic changes.
Provides protective barrier around the cell	Bacteria in aggregates were less preferred by grazers than freely suspended bacteria. EPS-producing deep-sea hydrothermal vent bacteria showed resistance to heavy metals. Metal binding involves cell wall components as well as polysaccharides. Exopolymer in sea-ice brine channels provided cryoprotection by interacting with water at low temperature to depress freezing point. Nutrient uptake by bacteria in aggregates was higher than for free-living cells in low nutrient systems.
Absorbs dissolved organic material	Porous and hydrated matrix acts like a sponge and sequesters and concentrates dissolved organics.

**Table 3:** Sugar and non sugar components of bacterial exopolysaccharides [151].

Type	Component	Example	Mode of Linkage
**Sugar**	Pentoses	d-Arabinose	
		d-Ribose	
		d-Xylose	
	Hexoses	d-Glucose	
		d-Mannose	
		d-Galactose	
		d-Allose	
		l-Ramnose	
		l-Fucose	
	Amino sugars	d-Glucosamine	
		d-Galactosamine	
	Uronic acids	d-Glucuronic acid	
		d-Galacturonic acid	
**Non sugar**	Acetic acid		*O*-acyl, *N*-acyl
	Succinic acid		*O*-acyl
	Pyruvic acid		Acetal
	Phosphoric acid		Ester, Diester
	Sulfuric acid		Ester
